# pH Level as a Marker for Predicting Death among Patients with *Vibrio vulnificus* Infection, South Korea, 2000–2011

**DOI:** 10.3201/eid2102.131249

**Published:** 2015-02

**Authors:** Na Ra Yun, Dong-Min Kim, Jun Lee, Mi Ah Han

**Affiliations:** Chosun University School of Medicine, Gwangju, South Korea

**Keywords:** Vibrio vulnificus, mortality, death, predictive marker, pH, APACHE score, bacteria

## Abstract

*Vibrio vulnificus *infection can progress to necrotizing fasciitis and death. To improve the likelihood of patient survival, an early prognosis of patient outcome is clinically important for emergency/trauma department doctors. To identify an accurate and simple predictor for death among *V. vulnificus*–infected persons, we reviewed clinical data for 34 patients at a hospital in South Korea during 2000–2011; of the patients, 16 (47%) died and 18 (53%) survived. For nonsurvivors, median time from hospital admission to death was 15 h (range 4–70). For predicting death, the areas under the receiver operating characteristic curves of the Acute Physiology and Chronic Health Evaluation (APACHE) II score and initial pH were 0.746 and 0.972, respectively (p = 0.005). An optimal cutoff pH of <7.35 had a sensitivity of 100% and specificity of 83%. Compared with the APACHE II score, the initial arterial blood pH level in *V. vulnificus-infected* patients was a more accurate predictive marker for death.

## Abstract 2

Initial pH level at hospital admission was the most accurate and simple predictor of death.

*Vibrio vulnificus* is a motile, halophilic, rod-shaped gram-negative pathogen that lives in estuarine environments (*1*). During the last decade, the prevalence of *V. vulnificus* infection has increased worldwide (*2*–*4*). In the United States, the annual number of *V. vulnificus *infections reported to the 10-state Foodborne Disease Active Surveillance Network (http://www.cdc.gov/foodnet/) increased from 0.01 cases/100,000 persons in 1996 to 0.05 cases/100,000 persons in 2010 (*5*).

*V. vulnificus* infections, which are mainly characterized by skin and soft-tissue infections or septicemia, can develop a fulminant course (*1*,*6*). The severe form of *V. vulnificus* soft-tissue infection, necrotizing fasciitis, often has adverse outcomes, including death. In such cases, death can occur within 48 h of hospital admission, especially if the infection is associated with the development of sepsis or septic shock, which increases the case-fatality rate to 26%–71% (*7*–*11*). For cases of *V. vulnificus* infection caused by the consumption of contaminated shellfish, the death rate is ≈53%, and the rate is higher (67%) among patients with liver disease (*12*). The death rate may increase to 100% in patients with septicemia if treatment is delayed for 72 h after symptom onset (*13*).

Surgery, including fasciotomy, debridement, and limb amputation, early in *V. vulnificus* infection has been advocated by some as a means for improving patient outcomes (*3*,*10*,*14*). However, the proper timing for surgery is debatable: in the early stages of infection, patients may not be stable enough to be moved to the operating room, and patients may have little chance of surviving if surgery is delayed until a later stage of infection (*3*). Because *V. vulnificus* infections can be life threatening, it is critical for emergency/trauma department doctors, including surgeons who make decisions regarding surgery for necrotizing fasciitis, to have early knowledge of a patient’s prognosis.

Some clinical data are available regarding the prognostic factors for patients with fatal *V. vulnificus* infection (*10*,*15*–*17*). Higher Acute Physiology and Chronic Health Evaluation (APACHE) II and Mortality in Emergency Department Sepsis (MEDS) scores have been reported to be significant prognostic indicators for *V. vulnificus*–infected patients (*10*,*15*). However, the APACHE II and MEDS scoring systems might be too complex to use quickly in an emergency setting. Thus, to find a marker to predict the risk for death among *V. vulnificus*–infected patients, we investigated the initial clinical and laboratory data available in hospital emergency departments. The aims of our study were to describe the clinical outcomes of *V. vulnificus*–infected patients and identify an accurate and simple predictive marker for death.

## Materials and Methods

### Patients

For the study, we identified 34 *V. vulnificus*–infected patients (>18 years of age) who had been admitted during January 2000–December 2011 to Chosun University Hospital, a tertiary teaching hospital in Gwangju, South Korea. *V. vulnificus* infection was diagnosed on the basis of blood and/or wound culture results. Clinical isolates were identified by using a Vitek II automated system (bioMérieux, Marcy l’Étoile, France). The study was approved by the Ethics in Human Research Committee of Chosun University Hospital.

### Data Collection and Definitions

Demographic information and data regarding patients’ clinical manifestations, laboratory variables, operations, antimicrobial drug regimens, underlying diseases, and time to death were obtained by chart review. In Chosun University Hospital, arterial blood gas analysis and blood cultures are routinely performed before starting empirical antimicrobial drug treatment in patients with signs or symptoms of infection.

The use of an appropriate antimicrobial drug regimen was noted if a patient had been administered ceftriaxone, cefotaxime, or ciprofloxacin alone or in combination with doxycycline. Chronic liver disease was noted if a patient had shown clinical or laboratory signs of chronic liver disease caused by alcoholism or by hepatitis B or C virus. Fasciotomy was noted if surgery had been performed for necrotizing fasciitis. Death was defined as dying while in the hospital.

### Statistical Analyses

We compared demographic and clinical characteristics for patients who survived and those who did not by using *t*-tests for continuous variables and χ^2 ^tests for categorical variables. For APACHE II scores and pH levels at admission, we calculated the areas under the receiver operating characteristic curves (AUROCs) for predicting death caused by *V. vulnificus *infection. Sensitivities, specificities, positive predictive values, negative predictive values, positive likelihood ratios (how much to increase the probability of survival if the test is positive), and negative likelihood ratios (how much to decrease the probability of survival if the test is negative) of various APACHE II scores and pH cutoff levels on admission were calculated (*18*). The Youden index was used to determine the optimal cutoff value (*19*).

Multivariate analyses were performed by using the Cox proportional hazards model; variables that showed a p value of <0.05 in univariate analyses were included in these analyses. Of the variables for arterial blood gas analysis, pH was the only variable included in the model, and the APACHE II score was excluded from the multivariate model because of collinearity with pH and creatinine. Survival curves were constructed by using the Kaplan–Meier method, and a log-rank test was used for comparison. Survival was measured from the time of hospital admission to the time of death. We evaluated death status as death occurring <120 h after admission; patients who lived >120 h after admission were not included in the death statistics. A 2-tailed p value of <0.05 was considered to indicate statistical significance. All statistical analyses were performed by using SAS software, version 8.2 (SAS Institute Inc., Cary, NC, USA).

## Results

### Clinical Outcomes for Patients with *V. vulnificus* Infection

Of the 34 patients with a diagnosis of *V. vulnificus* infection, 3 (9%) were female and 31 (91%) were male. A total of 25 patients had a history of consuming raw seafood, 5 had a wound exposure to seawater, and 4 were unaware of their exposure source. Of the 32 patients who had chronic liver disease, 27 (84%) had chronic alcoholism and 5 (16%) had hepatitis B virus infection.Of the 34 total patients, 16 (47%) died and 18 (53%) survived. The median time between arrival at the hospital and death was 15 h (range 4–70 h). For patients who survived and those who did not, the differences in underlying diseases, interval between symptom onset and hospital admission, initial appropriate antimicrobial drug treatment, and surgical treatment were not significant (Table 1).

**Table 1:** Demographic and clinical features for 34 patients in a study looking for predictors of death among persons with *Vibrio vulnificus *infection, South Korea, 2000–2011*

Variable	Nonsurvivors, n = 16†	Survivors, n = 18†	p value
Sex			0.346
M	15 (93.8)	15 (83.3)	
F	1 (6.3)	3 (16.7)	
Age, y	58 (43–74)	57 (45–67)	0.740
Interval, d, between symptom onset and hospital admission	3 (1–5)	3 (0–7)	0.304
Route of exposure			
Consumption of seafood	11 (69)	14 (78)	0.565
Wound exposure to seawater	3 (19)	2 (11)	0.723
Unknown	2 (12)	2 (22)	0.632
Underlying condition			
Chronic liver disease	15 (94)	17 (94)	0.872
Chronic alcoholism	13 (81)	14 (78)	0.803
Hepatitis B virus infection	2 (13)	3 (17)	0.732
Hepatitis C virus infection	0	0	NA
Signs and symptoms at hospital admission			
Any gastrointestinal symptom	7 (54.8)	5 (27.8)	0.331
Any skin and soft tissue lesion	12 (75.0)	15 (83.3)	0.549
Bacteremia present	10 (63)	8 (44)	0.292
Surgery performed	7 (44)	8 (44)	0.967
Appropriate antimicrobial drug treatment received	13 (81)	14 (78)	0.810

*NA, not applicable. †Except for age, data are numbers (%) or medians (range).

### Predictive Markers for Death

Univariate analysis showed that activated partial thromboplastin time and levels of pH, partial pressure of oxygen, serum bicarbonate, blood urea nitrogen, creatinine, aspartate transaminase, and alanine transaminase were significantly different between patients who survived and those who did not survive (Table 2). In multivariate analysis, initial pH on arterial blood gas analysis and serum creatinine level were significant risk factors for death (Table 3).

**Table 2:** Laboratory results 34 patients in a study looking for predictors of death among persons with *Vibrio vulnificus *infection, South Korea, 2000–2011*

Variable	Reference values	Values for patients		p value
		Nonsurvivors, n = 16	Survivors, n = 18	
Hematologic values				
Leukocyte count/μL	4,000–8,000	6,721 (1,030–25,950)	9,687 (1,940–21,770)	0.167
Hemoglobin, g/dL	12.0–16.0	12.5 (7.3–17.8)	11.5 (8.2–15.5)	0.248
Platelets, 10^3^/μL	150–400	62 (13–147)	81 (13–243)	0.330
PT, s	9.4–12.5	17.7 (10.5–30.4)	14.9 (10.2–19.5)	0.111
aPTT, s	28.0–44.0	48.5 (28.8–103.0)	37.4 (21.5–57.5)	0.040
Arterial blood gas analysis values				
pH	7.350–7.450	7.08 (6.82–7.36)	7.41 (7.22–7.50)	<0.001
HCO_3_, mmol/L	21.0–28.0	8.4 (3.6–13.9)	18.6 (8.7–25.6)	<0.001
PO_2_, mm Hg	83.0–108.0	103.4 (69.2–157.0)	86.6 (52.3–162.8)	0.035
PCO_2,_ mm Hg	35.0–45.0	26.7 (14.3–41.0)	28.8 (21.6–38.0)	0.314
Clinical chemistry values				
Albumin, g/dL	3.50–5.20	2.63 (2.07–3.00)	2.82 (1.66–3.84)	0.201
BUN, mg/dL	8.0–20.0	31.8 (10.0–56.9)	22.6 (9.6–49.7)	0.043
Creatinine, mg/dL	0.5–1.3	3.59 (1.52–5.90)	1.76 (0.60–3.90)	<0.001
Glucose, mg/dL	60–109	134 (22–306)	141 (40–300)	0.764
Bilirubin, mg/dL	0.2–1.2	7.5 (0.5–48.3)	2.3 (0.5–4.1)	0.094
Liver enzyme values				
AST, IU/L	5–40	443 (43–1,696)	111 (30–323)	0.006
ALT, IU/L	5–40	100 (22–290)	57 (17–133)	0.020
APACHE II score		18 (10–31)	14 (10–32)	0.036

*Data are expressed as median (range). ALT, alanine aminotransferase; APACHE, Acute Physiology and Chronic Health Evaluation; aPTT, activated partial thromboplastin time; AST, aspartate aminotransferase; BUN, blood urea nitrogen; HCO_3_, serum bicarbonate; PCO_2_, carbon dioxide partial pressure; PO_2_, partial pressure of oxygen; PT, prothrombin time.

**Table 3:** Prognostic factors for death, as determined by multivariate analysis, among patients with *Vibrio vulnificus* infection, South Korea, 2000–2011*

Variable	Relative risk (95% CI)	p value
pH (per 0.1 increase)	0.441 (0.305–0.637)	<0.001
Creatinine (per mg/dL increase)	2.114 (1.105–4.043)	0.023
BUN (per mg/dL increase)	1.046 (0.981–1.115)	0.171
AST (per IU/L increase)	0.998 (0.995–1.001)	0.221
ALT (per IU/L increase)	1.014 (0.991–1.039)	0.236
aPTT (/s increase)	0.983 (0.935–1.033)	0.496

*ALT, alanine aminotransferase; aPTT, activated partial thromboplastin time; AST, aspartate aminotransferase; BUN, blood urea nitrogen.

**Figure 1:**
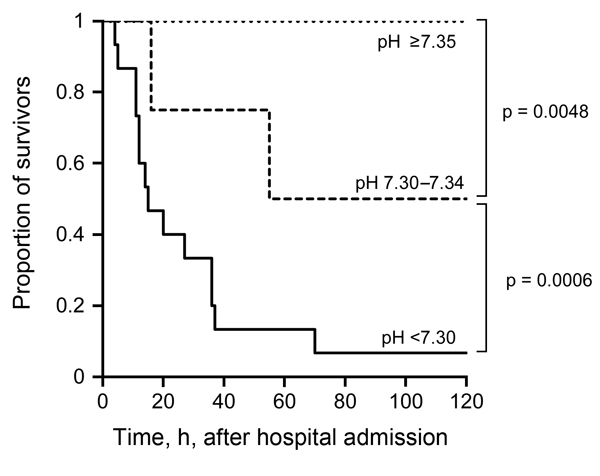
Survival curves of patients, by pH level at hospital admission, in a study investigating predictors of death among persons with *Vibrio vulnificus* infection, South Korea, 2000–2011.

**Figure 2:**
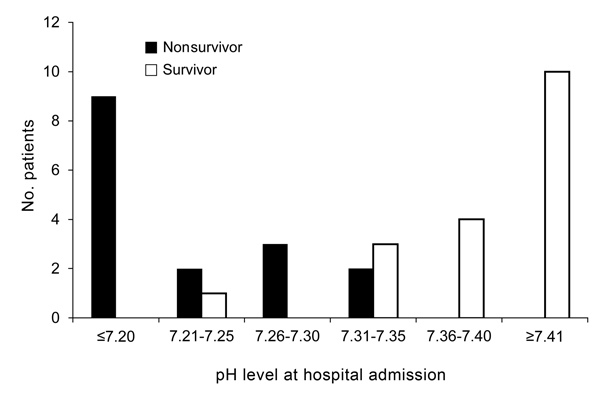
Numbers of surviving and nonsurviving patients, by pH level at hospital admission, in a study investigating predictors of death among persons with *Vibrio vulnificus* infection, South Korea, 2000–2011.

**Figure 3:**
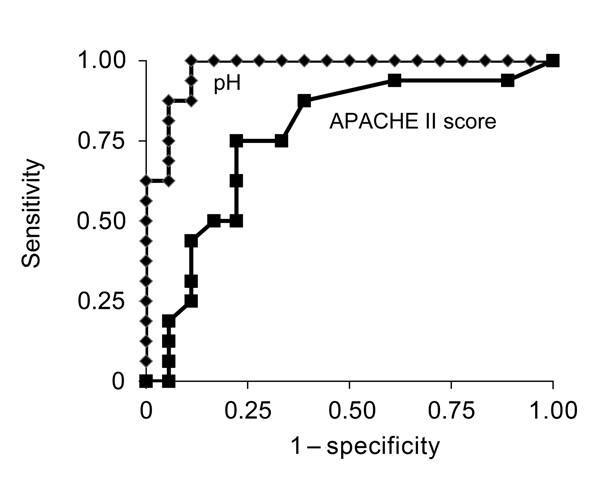
Receiver-operating characteristic curves (AUROCs) for pH level and Acute Physiology and Chronic Health Evaluation (APACHE) II score in a study investigating predictors of death among patients with *Vibrio vulnificus* infection, South Korea, 2000–2011. AUROC (95% CIs): pH level, 0.972 (range 0.924–1.000); APACHE II score,0.746 (range 0.595–0.933) (p = 0.005).

We found a significant difference in survival for patients with different arterial pH levels at hospital admission (Figure 1). No patients with a pH level of <7.2 at admission survived; all patients with a pH level of >7.35 survived (Figure 1, 2). An optimal pH cutoff level of <7.35 had a sensitivity of 100%, specificity of 83%, positive predictive value of 84%, negative predictive value of 100%, positive likelihood ratio of 6.0, and negative likelihood ratio of 0. An optimal APACHE II cutoff score of >14 had a sensitivity of 75%, specificity of 67%, positive predictive value of 67%, negative predictive value of 75%, positive likelihood ratio of 2.3, and negative likelihood ratio of 0.3 (Table 4). The AUROC of pH for prediction of death was 0.972 (95% CI 0.924–1.000), and the AUROC of the APACHE II score was 0.746 (95% CI 0.595–0.933) (p = 0.005) (Figure 3).

**Table 4:** Predictors of death among patients with *Vibrio vulnificus* infection, South Korea, 2000–2011*

Predictor	Sensitivity, %		Predictive value, %			Likelihood ratio	
		Specificity, %	Positive	Negative		Positive	Negative
pH level							
<7.30	87.5	94.4	93.3	89.5		15.63	0.13
<7.35	100.0	83.3	84.2	100.0		5.99	0
<7.40	100.0	61.1	69.6	100.0		2.57	0
APACHE II score							
>13	87.5	61.1	66.7	84.6		2.25	0.20
>14	75.0	66.7	66.7	75.0		2.25	0.37
>15	75.0	77.8	75.0	77.8		3.38	0.32

*APACHE, Acute Physiology and Chronic Health Evaluation.

## Discussion

In this study, approximately half of the patients with *V. vulnificus* infection died, and death occurred <72 h after hospital admission. Patient survival differed significantly by pH level at hospital admission, and the initial pH level for patients was a more accurate predictive marker for death than was the APACHE II score.

Many studies have asserted that antimicrobial drugs should be immediately administered to patients with suspected *V. vulnificus* infection (*3*,*8*,*15*,*20*). Other studies suggest that a combination of antimicrobial drugs (i.e., third-generation cephalosporin, ciprofloxacin, and doxycycline) is the best treatment for *V. vulnificus* infection (*21*,*22*). In addition, surgical intervention is necessary to remove necrotic tissue and bacteria (*20*). Survival of patients with necrotizing fasciitis has been shown to improve when adequate debridement and fasciotomy are performed early in infection (*3*,*20*,*23*,*24*).

However, in our study there was no difference in the time from symptom onset to hospital admission and the timing of the initiation of appropriate antimicrobial drug treatment and surgical treatment for patients who survived and those who did not. This lack of difference may partly be due to the fact that patients died too early after admission to benefit from antimicrobial drug treatment or surgery. The median time to death for nonsurviving patients in our study was 15 h, even though they arrived at the hospital relatively early after symptom onset. The time to death in our study seems to be shorter than that in another study, which showed 11 (73%) of the 15 deaths occurred <72 h after arrival at the hospital (*15*).

That earlier study and another study reported that APACHE II and MEDS scores at hospital admission were useful prognostic indicators in primary septicemia or wound infections caused by *V. vulnificus* (*10*,*15*). In another study, the level of *V. vulnificus* DNA was substantially higher in nonsurvivors than in survivors of *V. vulnificus* septicemia, and the *V. vulnificus* DNA load correlated with the APACHE II score (*17*). Our data also showed that the APACHE II score may be useful for prediction of death in *V. *vulnificus–infected patients, although the AUROC for the APACHE II score was significantly less than that for initial pH.

In previous reports, hemorrhagic bullae formation, necrotizing fasciitis, and septic shock were risk factors for death in patients with *V. vulnificus* infection (*8*,*25*–*27*). Although in our study, no skin or soft tissue lesions were associated with death, the pH and creatinine levels at hospital admission were independently associated with death of *V. vulnificus*–infected patients. Low arterial pH level might result from lactic acidosis caused by marked tissue hypoperfusion in shock and damage to extremities by necrotizing fasciitis (*28*,*29*). The finding that high creatinine level was associated with death also supports the possibility that hypoperfusion or sepsis contributed to the death of patients in this study because azotemia might result from renal hypoperfusion or septic renal injury.

Our study did have some limitations because of its retrospective design and the relatively small number of study patients, which may hamper generalization of the results. In addition, because most patients in this study had chronic liver disease, the study findings might not be applicable to patients with various concurrent diseases other than chronic liver disease.

Despite these limitations, our study may have relevant clinical implications. Our data demonstrate that the pH level determined by arterial blood gas analysis at hospital admission is more accurate than the APACHE II score as a marker for predicting death in *V. vulnificus*–infected patients. Arterial blood gas levels are easy to determine, and results are quickly available; thus, this test is useful in emergency or trauma departments.

 We propose that arterial pH level should be determined for patients with suspected *V. vulnificus* infection as soon as possible after hospital admission. When the arterial pH level is low (<7.2), patient outcome may be unsatisfactory, regardless of emergency surgery and appropriate antimicrobial drug therapy. When the pH level is not low, patient outcome may be excellent if the patient receives appropriate antimicrobial drug treatment and, if needed, surgery.

## References

[1] Chuang YC, Young CD, Chen CW. *Vibrio vulnificus* infection. Scand J Infect Dis. 1989;21:721–6. 10.3109/00365548909021703 2694352

[2] Okoh AI, Sibanda T, Nongogo V, Adefisoye M, Olayemi OO, Nontongana N. Prevalence and characterisation of non-cholerae *Vibrio* spp. in final effluents of wastewater treatment facilities in two districts of the Eastern Cape Province of South Africa: implications for public health. Environ Sci Pollut Res Int. 2014 [cited 2014 Nov19]. Epub 2014 Aug 29. 10.1007/s11356-014-3461-z PMC4308643 25167817

[3] Huehn S, Eichhorn C, Urmersbach S, Breidenbach J, Bechlars S, Bier N, Pathogenic vibrios in environmental, seafood and clinical sources in Germany. Int J Med Microbiol. 2014;•••:S1438–4221. 25129553 10.1016/j.ijmm.2014.07.010

[4] Lee YC, Hor LI, Chiu HY, Lee JW, Shieh SJ. Prognostic factor of mortality and its clinical implications in patients with necrotizing fasciitis caused by *Vibrio vulnificus.* Eur J Clin Microbiol Infect Dis. 2014;33:1011–8. 10.1007/s10096-013-2039-x 24419406

[5] Hsueh PR, Lin CY, Tang HJ, Lee HC, Liu JW, Liu YC, *Vibrio vulnificus* in Taiwan. Emerg Infect Dis. 2004;10:1363–8. 10.3201/eid1008.040047 15496235 PMC3320410

[6] Newton A, Kendall M, Vugia DJ, Henao OL, Mahon BE. Increasing rates of vibriosis in the United States, 1996–2010: review of surveillance data from 2 systems. Clin Infect Dis. 2012;54(Suppl 5):S391–5. 10.1093/cid/cis243 22572659 PMC4604744

[7] Chuang YC, Yuan CY, Liu CY, Lan CK, Huang AH. *Vibrio vulnificus* infection in Taiwan: report of 28 cases and review of clinical manifestations and treatment. Clin Infect Dis. 1992;15:271–6. 10.1093/clinids/15.2.271 1520762

[8] Howard RJ, Bennett NT. Infections caused by halophilic marine *Vibrio* bacteria. Ann Surg. 1993;217:525–31. 10.1097/00000658-199305010-00013 8489315 PMC1242837

[9] Liu JW, Lee IK, Tang HJ, Ko WC, Lee HC, Liu YC, Prognostic factors and antibiotics in *Vibrio vulnificus* septicemia. Arch Intern Med. 2006;166:2117–23. 10.1001/archinte.166.19.2117 17060542

[10] KuoChou TN. Chao WN, Yang C, Wong RH, Ueng KC, Chen SC. Predictors of mortality in skin and soft-tissue infections caused by *Vibrio vulnificus.* World J Surg. 2010;34:1669–75. 10.1007/s00268-010-0455-y 20151130

[11] Chen SC, Chan KS, Chao WN, Wang PH, Lin DB, Ueng KC, Clinical outcomes and prognostic factors for patients with *Vibrio vulnificus* infections requiring intensive care: a 10-yr retrospective study. Crit Care Med. 2010;38:1984–90 . 20657269 10.1097/CCM.0b013e3181eeda2c

[12] Tsai YH, Hsu RW, Huang KC, Huang TJ. Laboratory indicators for early detection and surgical treatment of vibrio necrotizing fasciitis. Clin Orthop Relat Res. 2010;468:2230–7. 10.1007/s11999-010-1311-y 20232179 PMC2895833

[13] Centers for Disease Control and Prevention. *Vibrio vulnificus* infections associated with raw oyster consumption—Florida, 1981–1992. MMWR Morb Mortal Wkly Rep. 1993;42:405–7 . 8497241

[14] Neupane GP, Kim DM. In vitro time-kill activities of ciprofloxacin alone and in combination with the iron chelator deferasirox against *Vibrio vulnificus.* Eur J Clin Microbiol Infect Dis. 2010;29:407–10. 10.1007/s10096-010-0875-5 20127132

[15] Halow KD, Harner RC, Fontenelle LJ. Primary skin infections secondary to *Vibrio vulnificus*: the role of operative intervention. J Am Coll Surg. 1996;183:329–34 . 8843261

[16] Chou TN, Lee YT, Lai YY, Chao WN, Yang C, Chen CC, Prognostic factors for primary septicemia and wound infection caused by *Vibrio vulnificus.* Am J Emerg Med. 2010;28:424–31. 10.1016/j.ajem.2008.12.037 20466220

[17] Hou CC, Lai CC, Liu WL, Chao CM, Chiu YH, Hsueh PR. Clinical manifestation and prognostic factors of non-cholerae *Vibrio* infections. Eur J Clin Microbiol Infect Dis. 2011;30:819–24. 10.1007/s10096-011-1162-9 21258834

[18] Kim DM, Jung SI, Jang HC, Lee CS, Lee H, Yun NR, *Vibrio vulnificus* DNA load and mortality. J Clin Microbiol. 2011;49:413–5. 10.1128/JCM.01913-09 21068289 PMC3020430

[19] Youden WJ. Index for rating diagnostic tests. Cancer. 1950;3:32–5. 10.1002/1097-0142(1950)3:1<32::AID-CNCR2820030106>3.0.CO;2-3 15405679

[20] Horseman MA, Surani S. A comprehensive review of *Vibrio vulnificus*: an important cause of severe sepsis and skin and soft-tissue infection. Int J Infect Dis. 2011;15:e157–66. 10.1016/j.ijid.2010.11.003 21177133

[21] Chen SC, Lee YT, Tsai SJ, Chan KS, Chao WN, Wang PH. etal. Antibiotic therapy for necrotizing fasciitis caused by *Vibrio vulnificus*: retrospective analysis of an 8 year period. J Antimicrob Chemother. 2012;67:488–93. 10.1093/jac/dkr476 22117030

[22] Jang HC, Choi SM, Kim HK, Kim SE, Kang SJ, Park KH. etal. Invivo efficacy of the combination of ciprofloxacin and cefotaxime against *Vibrio vulnificus* sepsis. PLoS ONE. 2014;9:e101118. 10.1371/journal.pone.0101118 24978586 PMC4076242

[23] Kuo YL, Shieh SJ, Chiu HY, Lee JW. Necrotizing fasciitis caused by *Vibrio vulnificus*: epidemiology, clinical findings, treatment and prevention. Eur J Clin Microbiol Infect Dis. 2007;26:785–92 . 10.1007/s10096-007-0358-5 17674061

[24] Chao WN, Tsai CF, Chang HR, Chan KS, Su CH, Lee T, Impact of timing of surgery on outcome of *Vibrio vulnificus*-related necrotizing fasciitis. Am J Surg. 2013;206:32–9. 10.1016/j.amjsurg.2012.08.008 23414632

[25] Klontz KC, Lieb S, Schreiber M, Janowski HT, Baldy LM, Gunn RA. Syndromes of *Vibrio vulnificus* infections. Clinical and epidemiologic features in Florida cases, 1981–1987. Ann Intern Med. 1988;109:318–23. 10.7326/0003-4819-109-4-318 3260760

[26] Chung PH, Chuang SK, Tsang T, Wai-man L, Yung R, Lo J. Cutaneous injury and *Vibrio vulnificus* infection. Emerg Infect Dis. 2006;12:1302–3. 10.3201/eid1208.051495 16972360 PMC3291212

[27] Penman AD, Lanier DC Jr, Avara WT III, Canant KE, DeGroote JW, Brackin BT, *Vibrio vulnificus* wound infections from the Mississippi Gulf coastal waters: June to August 1993. South Med J. 1995;88:531–3 . 10.1097/00007611-199505000-00004 7732441

[28] Madias NE. Lactic acidosis. Kidney Int. 1986;29:752–74 . 10.1038/ki.1986.62 3702227

[29] Mikkelsen ME, Miltiades AN, Gaieski DF, Goyal M, Fuchs BD, Shah CV, Serum lactate is associated with mortality in severe sepsis independent of organ failure and shock. Crit Care Med. 2009;37:1670–7 . 10.1097/CCM.0b013e31819fcf68 19325467

